# Supplementary material for: Role clarity deficiencies can wreck agile teams
Source: PeerJ Comput Sci. 2019 Dec 16;5:e241. doi: 10.7717/peerj-cs.241 (PMC7924487; doi:10.7717/peerj-cs.241)
Supplement: Supplemental Information 2 [file peerj-cs-05-241-s002.pdf]

Interview guideline (Original in German)

- Helena Barke, Ph.D. Student
- Interested in your personal perspective and experience as an expert
- Unusual situation: I will ask you a few open questions and you can just tell me in detail. You can decide for yourself what and how much. An interview takes about an hour.
- Open questions = methodology of Grounded Theory. Later maybe more detailed.
- I only listen and will not comment on anything. This is less like a natural dialogue / conversation. It's all about your perspective.
- If you are willing, I would like to record the interview. Of course everything is anonymized and strictly confidential. The interview is used for my Ph.D. and research work.

Icebreaker:

- What motivates you to work in Scrum teams?

Your team:

- In order to be able to follow your answers easier, can you please sketch your team on a piece of paper.

(ONLY if the interviewee still has no idea how to do it after some minutes: For example, as an organization chart: for each person, a circle and label with name or synonym and possibly scrum role or task.)

- Please tell me something about every team member.
  - **For properties like "shy" and so on : Why do you think that? Are there any situations you can tell me about?**
  - (How do the roles and tasks in your team look like?)
  - How do individual team members work together?
  - How is the relationship between the individual team members?

Collaboration in the team:

- Can you tell me about the last decision you made in the team?
- Can you tell me something about your user stories?
  - and how it is developed
  - and how is it processed?
  - can you tell me about a concrete example?

- Can you please tell me about a conflict in the team and how you solved it?
  - Do you usually solve your conflicts this way?

- How do you know that the team is doing well?
- How do you know that the team is not doing well?

Your person:

- (What do you like most or do not like?)
- (What competences would you like?)
- Can you tell me about a situation in which you have learned something from another person?
- Can you tell me about a situation in which another person has learned something from you?
- Anything else you want do add?

Many Thanks!
